# Novel biomarkers in LBD ‐ Implications for clinical trials

**DOI:** 10.1002/alz.089698

**Published:** 2025-01-09

**Authors:** Lisa Vermunt

**Affiliations:** ^1^ Alzheimer Center Amsterdam, Neurology, Vrije Universiteit Amsterdam, Amsterdam UMC location VUmc, Amsterdam, North Holland Netherlands

## Abstract

The lack of an in‐vivo pathology marker for synuclein pathology has been a long standing challenge for dementia for Lewy bodies (DLB) research. This issue is critically important for phase II trials, which are often small, requiring the precise measurement of the biological effects, whether disease modifying or symptomatic. Recent advances have enabled the determination of alpha‐synuclein pathology status with CSF measurements, using aggregation assays [RT‐QUIC]. Additionally, several proteomic studies have identified candidate markers for disease progression. This presentation focusses on these breakthroughs, and how they will influence selection of clinical trial participants and detection of treatment response. In clinical trial research, these markers could be instrumental in understanding the treatment effects in early stage trials (phase I‐II) and generate novel read‐outs for later stage trials (phase II‐III).

This talk will provide overview of novel CSF biomarkers, including Dopa Decarboxylase [DDC] and several others, exploring their potential implementation in clinical trials. The presentation will summarize converging evidence, offer forward‐looking perspectives, and present novel data on longitudinal cohort measurements for these markers, including DDC, to provide a comprehensive understanding of the potential applications of these biomarkers in the context of dementia research.

